# A 100-member ensemble simulations of global historical (1951–2010) wave heights

**DOI:** 10.1038/s41597-023-02058-6

**Published:** 2023-06-06

**Authors:** Mercè Casas-Prat, Xiaolan L. Wang, Nobuhito Mori, Yang Feng, Rodney Chan, Tomoya Shimura

**Affiliations:** 1 Climate Research Division, Science and Technology Branch, Environment and Climate Change Canada, Toronto, Ontario Canada; 2 Disaster Prevention Research Institute, Kyoto University, Kyoto, Japan

**Keywords:** Physical oceanography, Physical oceanography

## Abstract

The d4PDF-WaveHs dataset represents the first single model initial-condition large ensemble of historical significant ocean wave height (*H*_*s*_) at a global scale. It was produced using an advanced statistical model with predictors derived from Japan’s d4PDF ensemble of historical simulations of sea level pressure. d4PDF-WaveHs provides 100 realizations of *H*_*s*_ for the period 1951–2010 (hence 6,000 years of data) on a 1° × 1° lat.-long. grid. Technical comparison of model skill against modern reanalysis and other historical wave datasets was undertaken at global and regional scales. d4PDF-WaveHs provides unique data to understand better the poorly known role of internal climate variability in ocean wave climate, which can be used to estimate better trend signals. It also provides a better sampling of extreme events. Overall, this is crucial to properly assess wave-driven impacts, such as extreme sea levels on low-lying populated coastal areas. This dataset may be of interest to a variety of researchers, engineers and stakeholders in the fields of climate science, oceanography, coastal management, offshore engineering, and energy resource development.

## Background & Summary

Ocean wind-waves, hereafter called waves, are an important element of the climate system, modulating the interactions between the atmosphere and the oceans^1^. They are also a key environmental variable for coastal and offshore engineering^2^, and affect many coastal dynamics processes^3^, navigation planning^4^, and are a potential source of renewable energy^5^. Over 300 million people live in low-lying coastal areas^6^, and detailed knowledge of wave climate is essential to address the environmental and societal wave-driven impacts properly.

The IPCC (2013)^7^ highlighted a low knowledge confidence of wave climatology in comparison with many other climate variables. This relates to the fact that most climate models provide no information about waves; therefore, the availability of wave simulations is relatively limited. To fill in this gap, a growing number of studies have been developed over the last decade, producing several global and regional wave datasets. Most of these efforts were consolidated with COWCLIP2.0^8^, the first coherent, community-driven multi-method ensemble of historical and future global wave simulations, which included dominant sources of uncertainty, namely forcing uncertainty, and wave and climate model uncertainty. However, the internal climate variability was not properly sampled as most combinations of forcing and climate/wave models considered just one realization of the climate system. Studies based on a single (or reduced sample of) realizations of the climate system might underestimate extreme events or confound trends with internal climate variability^9,10^.

More recently, a global ensemble of ocean wave climate statistics from contemporary wave reanalysis and hindcasts^11^ highlighted the discrepancies among modern wave products of historical data. However, this database cannot provide insight into the role of the internal climate variability either due to the relatively short time period considered (1980–2014). Also, wave reanalysis/hindcasts are constructed to replicate the observed climate and, therefore, they all correspond to the same realization of the climate system.

The internal climate variability can be investigated with a Single Model Initial-condition Large Ensemble (SMILE), which is a set of simulations starting from different initial conditions but produced with a single climate model and identical external forcing^12^. Over the last decade, SMILEs have been increasingly generated and used in climate science as they represent very valuable data to study not just internal climate variability but also extremes^13,14^. The number of ensemble members required to obtain robust estimates depends on targets or temporal and spatial averaging scale^15^. However, most studies conclude that single realizations are insufficient to assess climate statistics and that large samples (size of >20–30 members) are required^10^. However, as it happens for other ensembles produced by climate models, existing SMILEs do not provide information about waves.

Here, we present and describe d4PDF-WaveHs, the first SMILE-based ensemble of global significant wave height (*H*_*s*_) simulations. *H*_*s*_ is a well-defined and standardized statistic to describe the characteristic wave height of the sea state, which is defined as the average height of the highest one-third of waves, and it is largely used in coastal, naval, and offshore engineering. d4PDF-WaveHs was produced with an advanced statistical model^16,17^ and using d4PDF’s historical simulations of sea level pressure (SLP) developed by the Japan Meteorological Research Institute with the MRI-AGCM atmospheric global climate model^18^. This dataset is archived in Network Common Data Form (NetCDF) with CF (Climate & Forecasts) compliant metadata, and contains 100 realizations of global *H*_*s*_ simulations over the period 1951–2010 on a 1° spatial grid resolution. The dataset provides a variety of standard *H*_*s*_ global statistics at monthly, seasonal, and annual time scale, as well as a set of extreme *H*_*s*_ indices designed by the Expert Team on Climate Change Detection (ETCCDI), using a standardized framework as in COWCLIP2.0 (see Tables 1, 2).

**Table 1:** d4PDF-WaveHs set of *H*_*s*_ statistics (via *getStat.f*).

Statistics ID	Indicator name	Time-frame resolution	Units
Hs_avg	Mean significant wave height	Annual (1), Seasonal (4) and Monthly (12)	m
Hs_p10	10th Percentile significant wave height	Annual (1), Seasonal (4) and Monthly (12)	m
Hs_p50	50th Percentile significant wave height	Annual (1), Seasonal (4) and Monthly (12)	m
Hs_p90	90th Percentile significant wave height	Annual (1), Seasonal (4) and Monthly (12)	m
Hs_p95	95th Percentile significant wave height	Annual (1), Seasonal (4) and Monthly (12)	m
Hs_p99	99th Percentile significant wave height	Annual (1), Seasonal (4) and Monthly (12)	m
Hs_max	Maximum significant wave height	Annual (1), Seasonal (4) and Monthly (12)	m

**Table 2:** d4PDF-WaveHs set of *H*_*s*_ statistics (via *getHsEx.f*).

Statistics ID	Indicator name	Definition	Units
HsRo	Rough wave days	Annual count of days when daily max *H*_*s*_ > 2.5 m	days
HsHi	High wave days	Annual count of days when daily max *H*_*s*_ > 6 m	days
fHsRo	Frequency of rough wave days	Annual percentage of days when daily max *H*_*s*_ > 2.5 m	%
fHsHi	Frequency of high wave days	Annual percentage of days when daily max *H*_*s*_ > 6 m	%
fHs10p	Frequency of top decile wave days	Annual percentage of days when daily max *H*_*s*_ > 10th percentile of daily max *H*_*s*_ in base period*	%
fHs90p	Frequency of top decile wave days	Annual percentage of days when daily max *H*_*s*_ < 90th percentile of daily max *H*_*s*_ in base period*	%
HHsDI	Top decile wave spell duration indicator	Annual count of days with at least 2 consecutive days when daily max *H*_*s*_ > 90th percentile of daily max *H*_*s*_ in the base period*	days

*Base period is 1980–2010.

The d4PDF-WaveHs dataset provides valuable data that can be used to advance understanding of the poorly known role of the internal wave climate variability. This can result in a more robust assessment of *H*_*s*_ trends and low-frequency extremes. For instance, the annual set of wave statistics from the d4PDF-WaveHs ensemble was recently used to quantify the role of the internal climate variability (in comparison to other uncertainty factors) in the assessment of the annual mean and maximum *H*_*s*_ trends^19^. d4PDF-WaveHs can also contribute to improve broad-scale coastal hazard and vulnerability assessments. This extensive wave information can now be widely used by different stakeholders, engineers, and research communities, such as those focusing on natural hazards, coastal management, port, and offshore engineering, energy resource development and ship navigation.

## Methods

This section describes the methodology used to generate the original sub-daily data, and the post-processed statistics and indices, which follows a standardized framework.

### Generation of original sub-daily data

The modelling approach of Wang *et al*.^16,17^ was used to produce d4PDF-WaveHs. The main aspects of this advanced statistical model are summarized below but more details can be found in the reference papers^16,17^ and in the derived study that assesses trend uncertainty^19^. For each grid point of a global 1°× 1° lat.-long. grid, a multivariate regression model with lagged dependent variable was developed. This regression model consists of expressing 6-hourly *H*_*s*_ as a function of 6-hourly anomalies (relative to the 1981–2000 mean) of SLP and of squared SLP gradients at each grid point, as well as the 30 leading principal components (PCs) of the SLP and of spatial SLP gradients fields over a large area of influence. 13 modelling regions were used (see Figure S1), which represents a slight modification of the original approach^16^, which used 11 modelling regions (ETNP and WTNP were a single region, as well as ETSP and WTSP). For each of these 13 regions a different set of PCs is considered. The consideration of local data accounts for local geostrophic wind energy, which drives local wind-sea states, while the set of PCs describe the large scale patterns of atmospheric circulation that affect remotely generated (swell) waves arriving at a target grid point. In total, for each wave grid point, the model uses a pool of 62 potential SLP-based predictors and employs the *F* test with the equivalent sample size^20^ to determine which and how these potential predictors are retained. Additionally, a Box-Cox power transformation^21^ is applied to both *H*_*s*_ and the predictors to minimize their departure from a normal distribution.

As in Wang *et al*.^17^, the European Center for Medium-Range Weather Forecasts (ECMWF) Reanalysis Interim (ERAI)^22^ was used to calibrate the model and bias-correct the predictors for each of the four seasons^17^. Particularly, the correction of predictors accounts for adjusting SLP fields to have the same climatological mean and standard deviation as ERAI SLP data. Additionally, any simulated *H*_*s*_ values that exceed twice the largest *H*_*s*_ from ERAI for a given season are excluded (set to missing). This cap is needed as very rarely (0.05‰ in all simulated *H*_*s*_ data) the Box-Cox transformation of the SLP gradients leads to an overgrowth of sharp SLP gradients that causes unrealistic *H*_*s*_ values. We used ERAI as calibration dataset in order to be consistent with previous studies and, in particular, the ECCC(s) sub-ensemble of the COWCLIP2.0 dataset^8^. This enables a better comparison between the role of the internal climate variability and model uncertainty (e.g. for trend assessment^19^). However, considering the higher resolution and methodological advances implemented in the newer ERA5 dataset^23^, we acknowledge that future studies should consider the re-calibration of the statistical model using ERA5.

To generate d4PDF-WaveHs, the aforementioned SLP-based predictors were obtained from the historical simulations of Japan’s d4PDF large ensemble (which contains simulations of historical and future climates)^18^. d4PDF was obtained with the 60-km resolution MRI-AGCM model^24^ developed by the Japan Meteorological Research Institute. The historical d4PDF SMILE-type ensemble used in this study was generated by perturbations of the historical sea surface temperature, sea ice concentration, and sea ice thickness in relation to the observed errors, while using the same forcing and global mean concentration of greenhouse gases. The d4PDF dataset (available at https://diasjp.net/en/service/d4pdf-data-download) satisfactorily simulates the past climate in terms of climatology, natural variations, and extreme events; and has been used in more than 70 papers^15^.

### Computation of statistics and indices

**Fig. 1:**
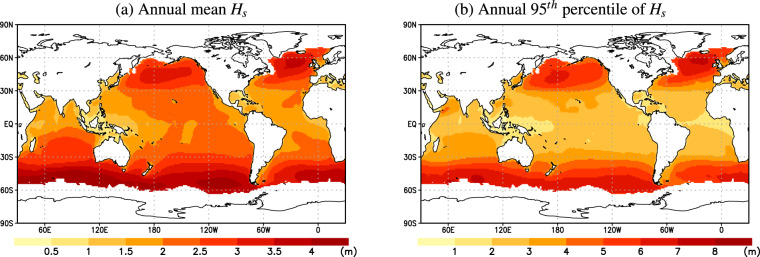
Ensemble average of the annual mean *H*_*s*_ and 95^*th*^ percentile of *H*_*s*_ climatological mean (m) as obtained from d4PDF-WaveHs for 1951–2010.

To calculate the *H*_*s*_ statistics and indices for each ensemble member of the 6-hourly *H*_*s*_ simulations, we used a standardized framework similar to the protocol used for the COWCLIP2.0 dataset. The original sub-daily *H*_*s*_ data was post-processed with the COWCLIP Fortran code *getStat.f* and *getHsEx.f*^25^ (after a slight modification to allow for missing data). We thus obtained a standard set of wave statistics (at annual, seasonal, and monthly time-frame resolutions). With *getStat.f*, we calculated seven wave *H*_*s*_ statistics: mean, 10th, 50th, 90th, 95th, 99th percentiles, and maximum (see Table 1). The seasonal statistics were computed on default seasons defined as DJF (December-January-February), MAM (March-April-May), JJA (June-July-August), and SON (September-October-November). Note that the d4PDF-WaveHs ice-covered areas were kept the same as those corresponding to ERAI. As an example, Figure 1 shows the 1951–2010 climatological mean of the annual mean and 95^*th*^ percentile of *H*_*s*_, as derived from the d4PDF-WaveHs ensemble.

The *getHsEx.f* was used to calculate an ETCCDI set of extreme annual *H*_*s*_ indices, using the baseline period over 1980–2010 to compute the relative statistics. The output netCDF contains the following seven extreme statistics calculated annually: rough wave days, high wave days, frequency of high wave days, frequency of top (low) decide wave days, frequency of top (high) decide wave days, top decile wave spell duration indicator (see Table 2).

The data obtained with the Fortran code described above was post-processed with standard NetCDF operators (NCOs) for file manipulation, such as the “ncatted” command, to include all relevant metadata, including variables’ attributes: “long_name” and “units”, as well as several global attributes about the project, modelling centres, forcing and experiments configuration.

## Data Records

The full archived dataset^26^ comprising the statistics and indices described above (consult Methods section) can be accessed through the Open Data portal of the Government of Canada (https://open.canada.ca/), at DOI: 10.18164/d68361d0-8141-48b9-a25e-a9bc98d71438.

The data set in total comprises 400 files, with a total volume of 184 GB. We used a consistent directory structure and file naming with the following Data Reference Syntax (DRS):

- Directories: *d4PDF*-*WaveHs*/*historical*/<*ensemble_member*>/<*version*>/<*frequency*>
- Filenames: *Hs*_*glob_d4PDF-WaveHs_historical*_<*ensemble*_*member*>_<*frequency*>_*1951–2010.nc*

where <*ensemble_member*> is of the form “r*N*”, being *N* the corresponding ensemble member that goes from 1 to 100. <*version*> is given in the form “vYYYYMM” (year/month) and <*frequency*> can be “ann”, “seas” or “mon”.

Recommended global attributes were defined and included accounting for the Attribute Convention for Dataset Discovery (ACDD) standards compliance. Note that although we followed many of the CF convention guidelines, the files are not strictly CF-compliant in time dimension - which uses units “years since” and “months since” the reference date, but this is consistent with the COWCLIP2.0 dataset^8^.

## Technical Validation

The statistical modelling approach used to generate the sub-daily data in this study has been used and validated in several previous studies at global and regional scales, and to assess trends, projected changes, and variability^16,17,27^. It was also used to generate the ECCC(s) sub-ensemble of the COWCLIP2.0 dataset^8^, which has led to improved understanding of the historical and future wave climates^28,29^. Moreover, the d4PDF dataset used to compute the predictors has been extensively examined and assessed for model skill against satellite observations and reanalysis datasets in several studies^15^.

Additionally, the resulting model-skill of the presented d4PDF-WaveHs dataset was compared against similar global wave simulations and modern reanalysis. In particular, we considered the CMIP5-driven historical simulations of COWCLIP2.0 (CMIP5-COWCLIP)^8^, with special emphasis on the ECCC(s) sub-ensemble (CMIP5-ECCC(s)), and the global ensemble of ocean wave climate statistics from contemporary wave reanalysis and hindcasts^11^. The latter includes 2 wave reanalysis (ERAI^22^, ERA5^23^) and 12 wave hindcasts that are driven by 6 wind reanalysis products (for more details refer to Morim *et al*.^11^):

- NCEP/NCAR-driven products: NCEPNCAR-IHC-GOW1.0^30^
- CFSR-driven products: CFSR-CSIRO-G1D^31^, CFSR-CSIRO-CAWCR^32^, CFSR-IHC-GOW2.0^33^, CFSR-JRC^34^, CFSR-IFREMER^35^
- ECMWF ERAI-driven products: ERAI-JRC^34^, ERAI-NOC^36^
- ECMWF ERA5-driven products: ERA5H^37,38^
- JMA JRA-55-driven products: JR55-KU-ST2^39,40^, JRA55-KU-ST4^39,40^
- MERRA2-driven products: MERRA2-IORAS^41^

Additionally, we compared the first ensemble member of d4PDF-WaveHs with the corresponding *H*_*s*_ obtained with the traditional dynamical modelling approach. Specifically, the WAVEWATCH III version 5 with a spatial resolution of 0.5625°^42^ was run with the surface winds of the first d4PDF member. All datasets were compared altogether for the common period between 1980 and 2004.

The model performance was assessed in terms of the climatology distribution rather than the replication of particular weather events because climate simulations are not in phase with observations. In particular, the normalized version of Taylor diagram^43^ was used for technical validation, which provides information about the spatial correlation, normalized standard deviation and normalized centred-root-mean-square difference of the *H*_*s*_ statistic of the model in question relative to the corresponding value of the reference dataset (the statistics were normalized, and non-dimensionalized, dividing by the standard deviation of the reference dataset). Since Taylor diagrams do not provide bias information, the technical validation also included comparison of the root-mean-square difference vs. the absolute difference (also relative to the reference dataset). The most recent reanalysis, ERA5, was used as reference dataset for both the Taylor diagrams and bias plots. Note this choice was made for comparison purposes only with the main goal to show the performance of the d4PDF-WaveHs in comparison to the variability among modern wave data products.

**Fig. 2:**
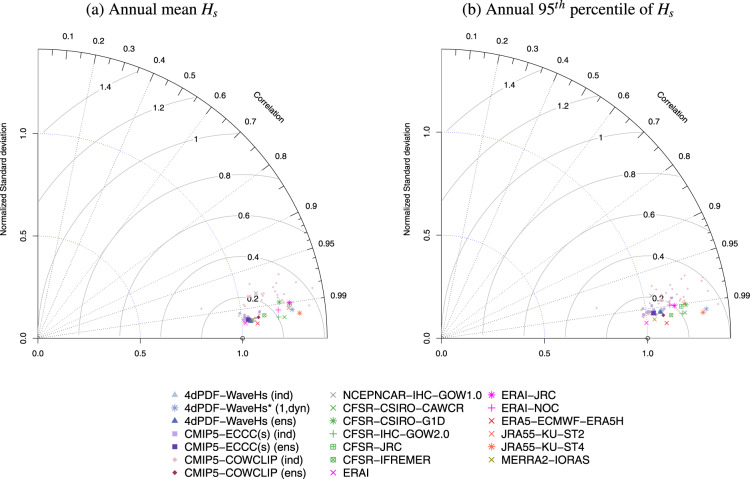
Normalized Taylor Diagram for the annual mean *H*_*s*_ (**a**) and the annual 95^*th*^ percentile of *H*_*s*_ (**b**) mean climatologies of the indicated wave products using ERA5 as reference. Normalized standard deviation is on the radial axis, correlation coefficient is on the angular axis and the gray lines indicate the normalized centred-root-mean-square difference.

**Fig. 3:**
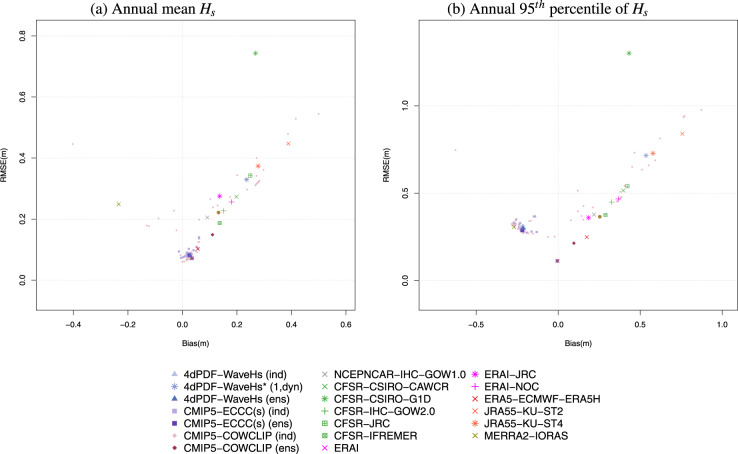
RMSE (m) vs. Bias (m) for the annual mean *H*_*s*_ (**a**) and the annual 95^*th*^ percentile of *H*_*s*_ (**b**) mean climatologies of the indicated wave products using ERA5 as reference.

Figures 2, 3 show the performance of the annual mean and the annual 95^*th*^ percentile of *H*_*s*_ climatologies at global scale. Additional results at regional scale are provided in the Supplementary Material (Figures S2–S21). For the ensemble of historical simulations, both the ensemble members and the ensemble average are illustrated in lighter and darker shades, respectively, of the same colour. Also, the wave hindcasts/reanalysis are grouped (in colour) in relation to the driving wind reanalysis. As expected, the performance of the global annual mean *H*_*s*_ obtained by d4PDF-WaveHs ensemble is similar to ECCC(s) and ERAI (Fig. 2a), as the later was used in the calibration and predictors’ adjustment step of the statistical methodology used to simulate d4PDF-WaveHs and ECCC(s). These performances are also close to ERA5 with a relatively low RMSE and bias in comparison to the other wave products used for comparison. The largest RMSE is obtained for CFSR-CSIRO-G1D but the largest biases are obtained for a few CMIP5-COWCLIP members (Fig. 3a). For the annual 95^*th*^ percentile of *H*_*s*_, d4PDF-WaveHs tends to underestimate the corresponding ERA5 values but the overall performance is good in comparison to the overall uncertainty of all wave products (see Figs. 2b, 3b). Also, this negative bias is reduced for mid to high latitudes.

**Fig. 4:**
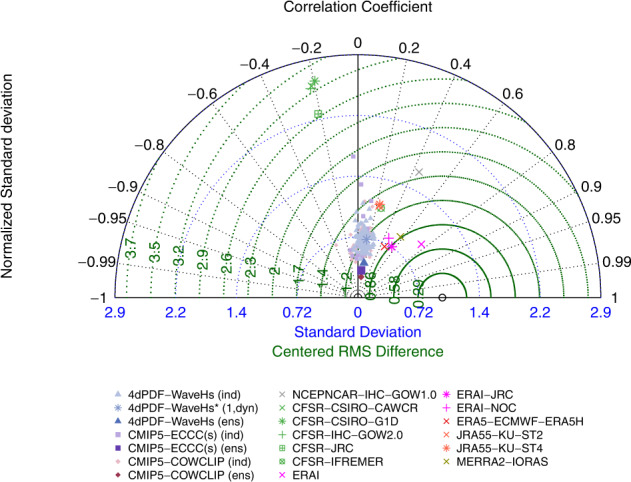
Normalized Taylor Diagram for the annual mean *H*_*s*_ trend of the indicated wave products using ERA5 as reference. Normalized standard deviation is on the radial axis, correlation coefficient is on the angular axis and the dotted green lines indicate the normalized centred-root-mean-square difference. Note that the second quadrant on the left indicates negative correlations.

As expected, the internal climate variability (as described here by the d4PDF-WaveHs inter-member variability) of the climatological mean *H*_*s*_ is very small. A slightly larger spread is obtained for the 95^*th*^ percentile, which further increases at regional scales (Figs. S3–S7). This inter-member variability is expected to further increase for lower frequency events. This is because the assessment of extremes is more sensitive to the internal climate variability than the average climate. The internal climate variability also plays an important role in the trend analysis. Figure 4 shows how the spread of trends of the annual mean *H*_*s*_ corresponding to the d4PDF-WaveHs members is comparable to the variability of the corresponding values for the COWCLIP dataset, which includes the uncertainty derived from climate models and wave modelling approaches. Another interesting result from Figure 4 is the large disparity among reanalysis in terms of trends, being for example most CFSR-driven products negatively correlated with ERA5, which means that these datasets are exhibiting opposite patterns of trends. Spatial correlation among products is in general medium to low for most products with a correlation lower than 0.6 (the correlation is between 0 and 0.2 for d4PDF-WaveHs members). These discrepancies have been noted in previous studies and are arguably related to the changes in data assimilation over time in reanalysis products^19,35,41^.

Overall, the d4PDF-WaveHs performance is reasonable given the uncertainty among modern wave data products. The mean climatology is comparable to that obtained from modern reanalysis while extremes tend to be underestimated (especially in tropics), as similarly obtained for the other historical simulations used here for comparison that do not consider data assimilation (CMIP5-COWCLIP, including CMIP5-ECCC(s)). It is particularly challenging to assess the performance of trends given the existing discrepancies among reanalysis. Further data validation is advisable to assess other wave features.

## Usage Notes

The data can be used with a wide range of postprocessing software, such us Ferret or NCL, and several packages from Python, R or MATLAB among others.

## Supplementary information

Supplementary Material

## Data Availability

**Sub-daily data generation code**The technical details of the statistical model used to generate the 6-hourly *H*_*s*_ from SLP predictors are included in the corresponding reference paper^16^ which allow for the reproducibility of the presented dataset. Additionally, the corresponding Fortran and R codes are publicly available in the Government of Canada Open Data Portal, together with the d4PDF-WaveHs dataset (DOI 10.18164/d68361d0-8141-48b9-a25e-a9bc98d71438). **Computation of statistics/indices:** ***getStat.f***, ***getHsEx.f***To be consistent with COWCLIP2.0^8^, the *H*_*s*_ statistics and indices were computed with *getStat.f* and *getHsEx.f*, after a slight modification to account for missing data (see Methods Section). The original Fortran code^25^ was developed as part of the COWCLIP community framework and can accessed via the COWCLIP website (https://cowclip.org/data-access). The code can be compiled with a Fortran compiler, with netCDF4 and HDF5 libraries. Additional attribute information to account for CF conventions and ACCD standards, was added to this Fortran code output with standard NetCDF operators (NCOs) for file manipulation, such as the “ncatted” command.
