# Supplementary material for: A 100-member ensemble simulations of global historical (1951–2010) wave heights
Source: Sci Data. 2023 Jun 6;10:362. doi: 10.1038/s41597-023-02058-6 (PMC10244329; doi:10.1038/s41597-023-02058-6)
Supplement: Supplementary file 1 — Supplementary Material [file 41597_2023_2058_MOESM1_ESM.pdf]

Supplementary material of "A 100-member ensemble simulations of global historical (1951–2010) wave heights"

Mercè Casas-Prat[1,*], Xiaolan L. Wang[1], Nobuhito Mori[2], Yang Feng[1], Rodney Chan[1], and Tomoya Shimura[2]

[1]Climate Research Division, Science and Technology Branch, Environment and Climate Change Canada, Toronto, Ontario, Canada
[2]Disaster Prevention Research Institute, Kyoto University, Japan
[*]corresponding author: Mercè Casas-Prat (merce.casasprat@ec.gc.ca)

Figures

Included here are Supplementary Figures S1-S21 that were referred to in the manuscript.

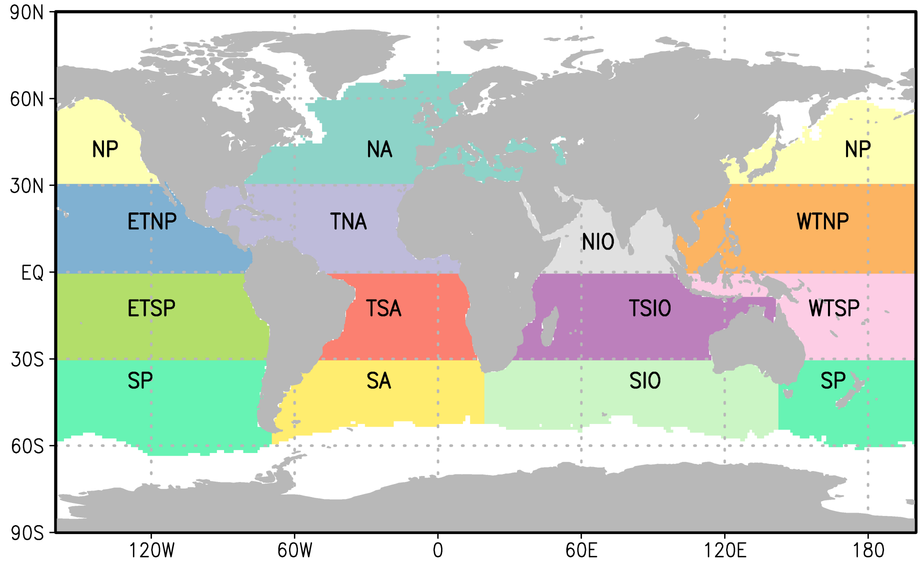

Figure S1. Areas used to model H_s and to compute the regional performance statistics.

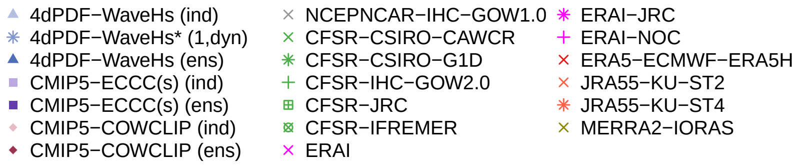

Figure S2. Legend for Figures S3-S21.

(a) Annual mean H_s (b) Annual 95th percentile of H_s

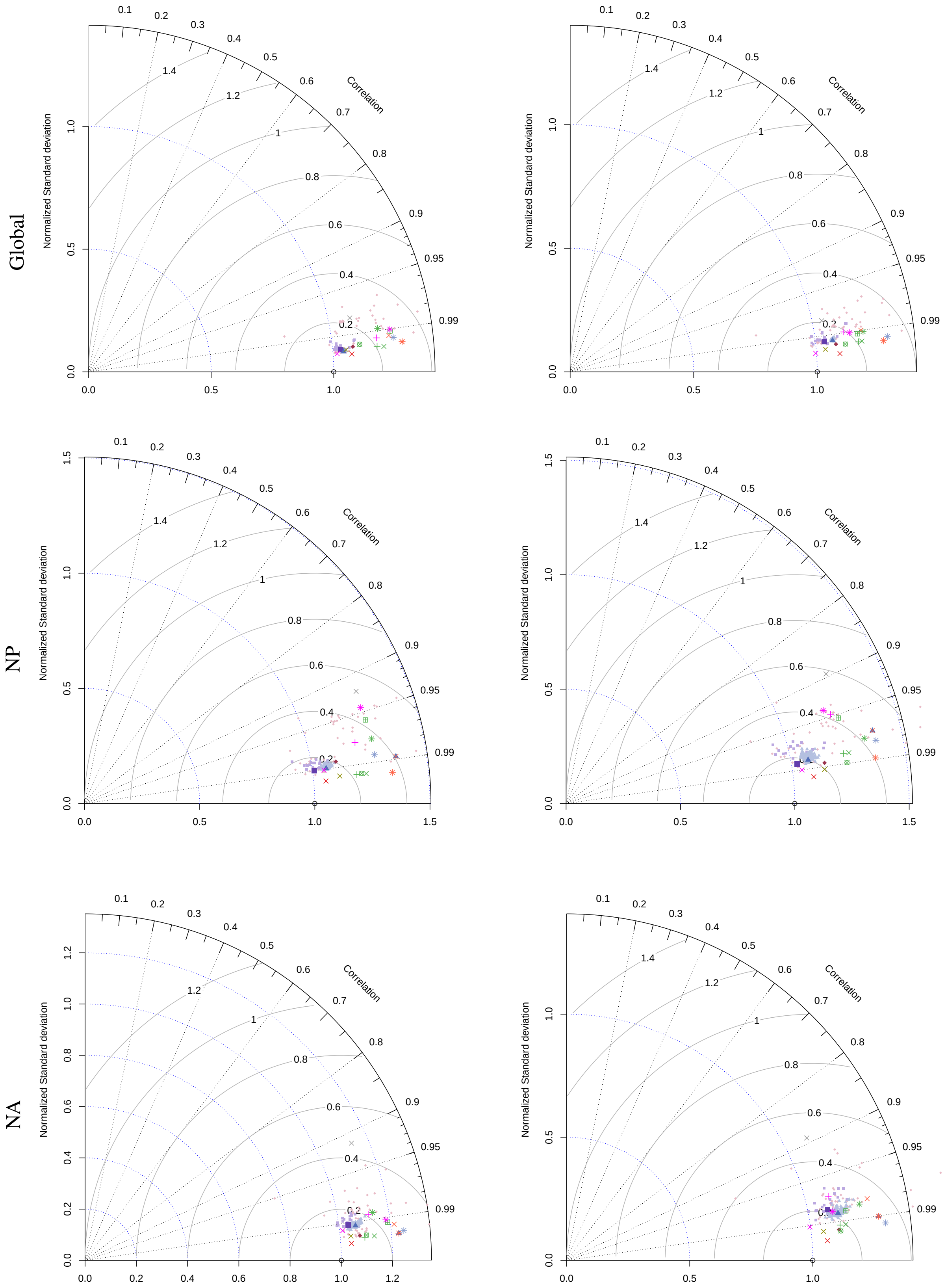

Figure S3. Normalized Taylor diagram for the climatological mean of the annual mean (a) and the annual 95th percentile (b) of H_s for the indicated regions (see Figure S1). ERA5 is used as reference. Legend shown in Figure S2.

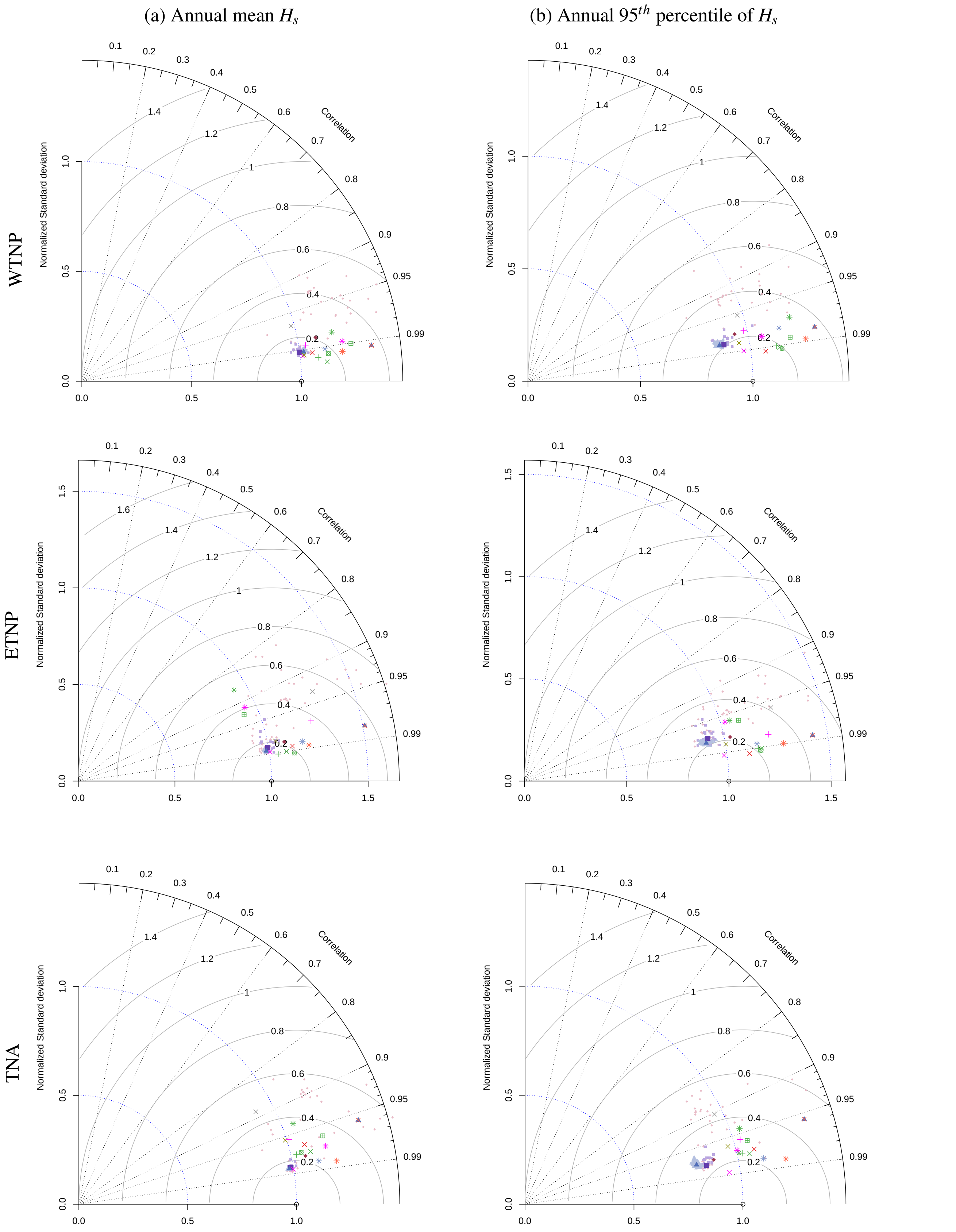

Figure S4. Normalized Taylor diagram for the climatological mean of the annual mean (a) and the annual 95th percentile (b) of H_s for the indicated regions (see Figure S1). ERA5 is used as reference. Legend shown in Figure S2.

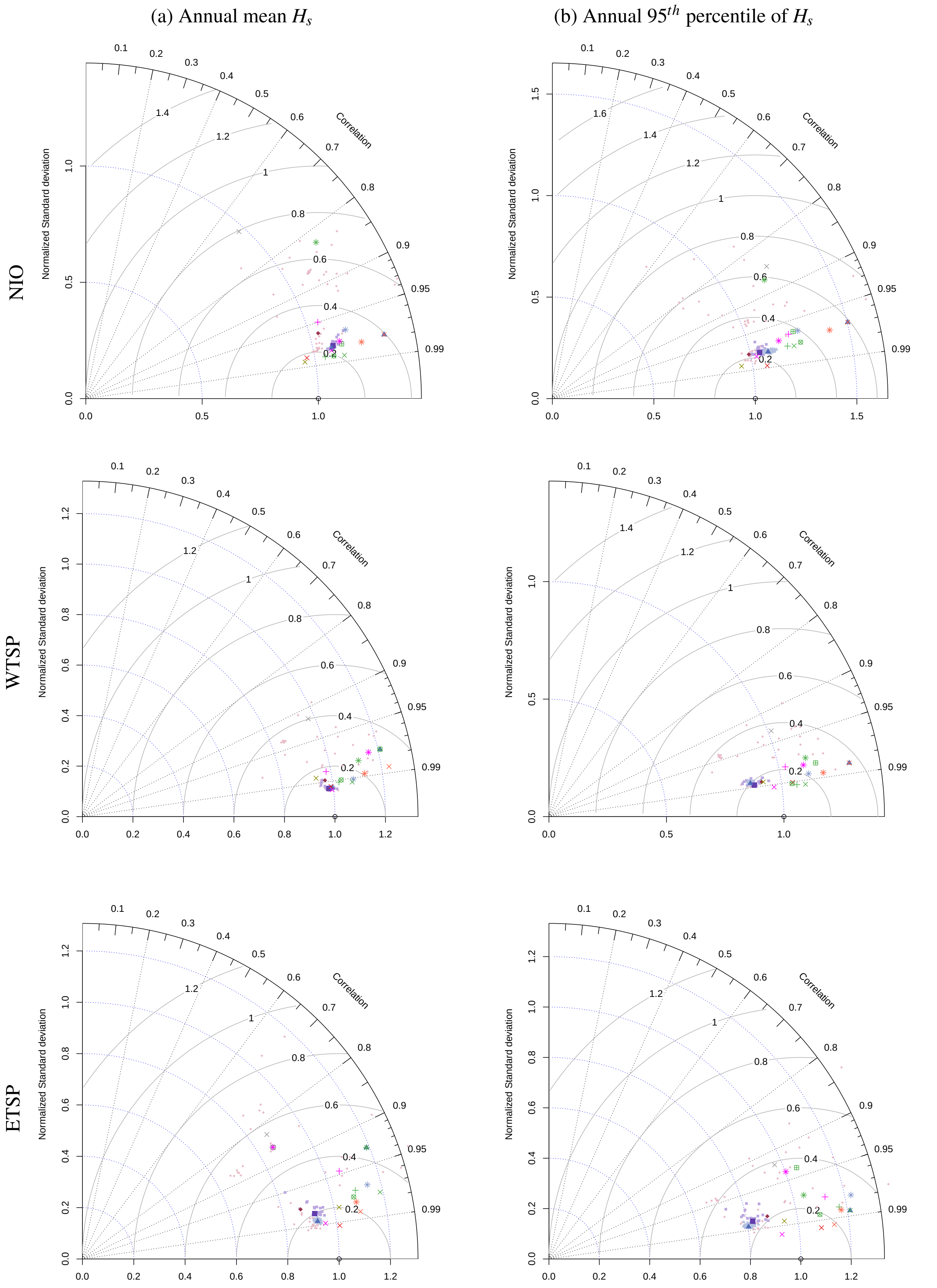

Figure S5. Normalized Taylor diagram for the climatological mean of the annual mean (a) and the annual 95th percentile (b) of H_s for the indicated regions (see Figure S1). ERA5 is used as reference. Legend shown in Figure S2.

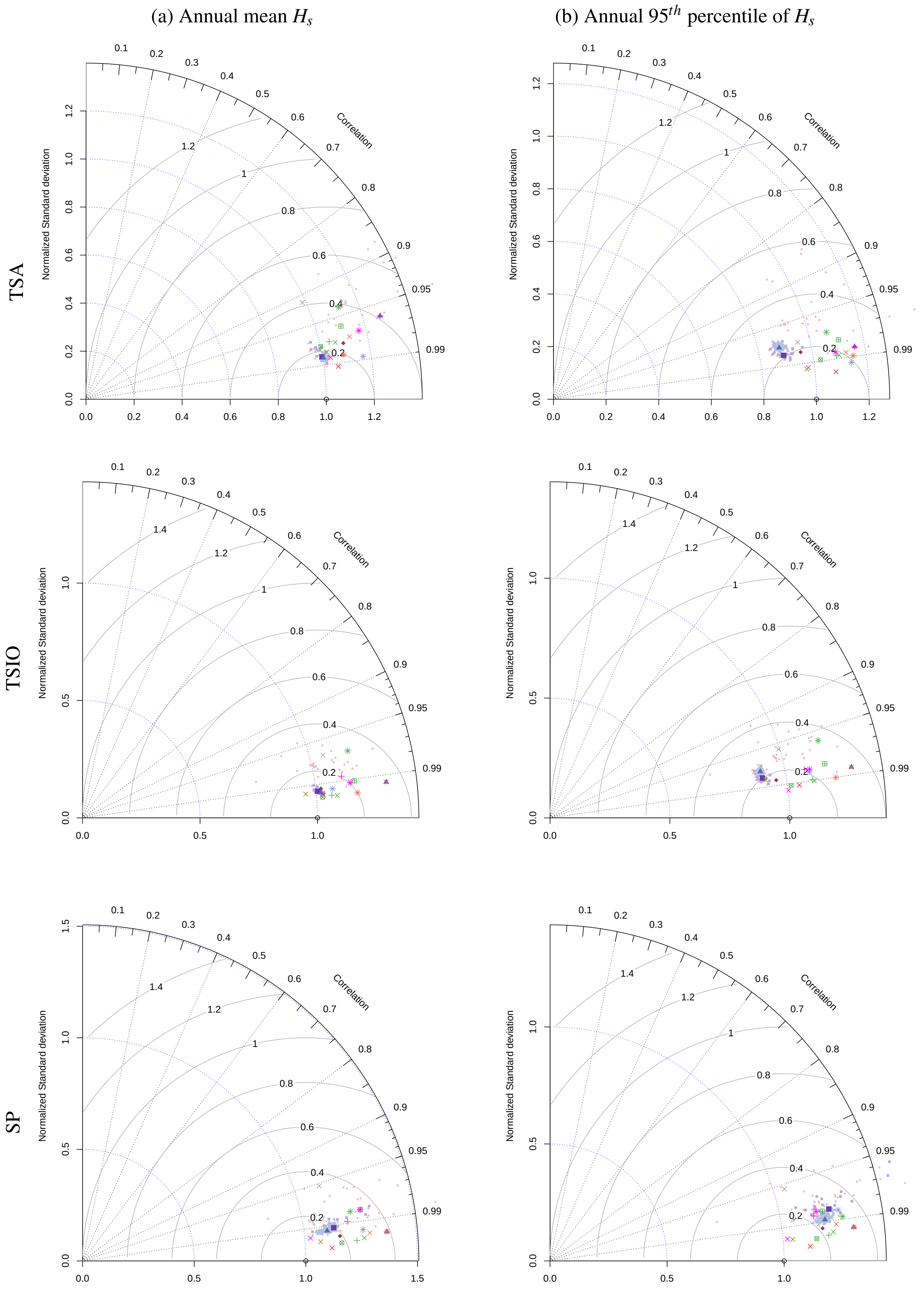

Figure S6. Normalized Taylor diagram for the climatological mean of the annual mean (a) and the annual 95th percentile (b) of H_s for the indicated regions (see Figure S1). ERA5 is used as reference. Legend shown in Figure S2.

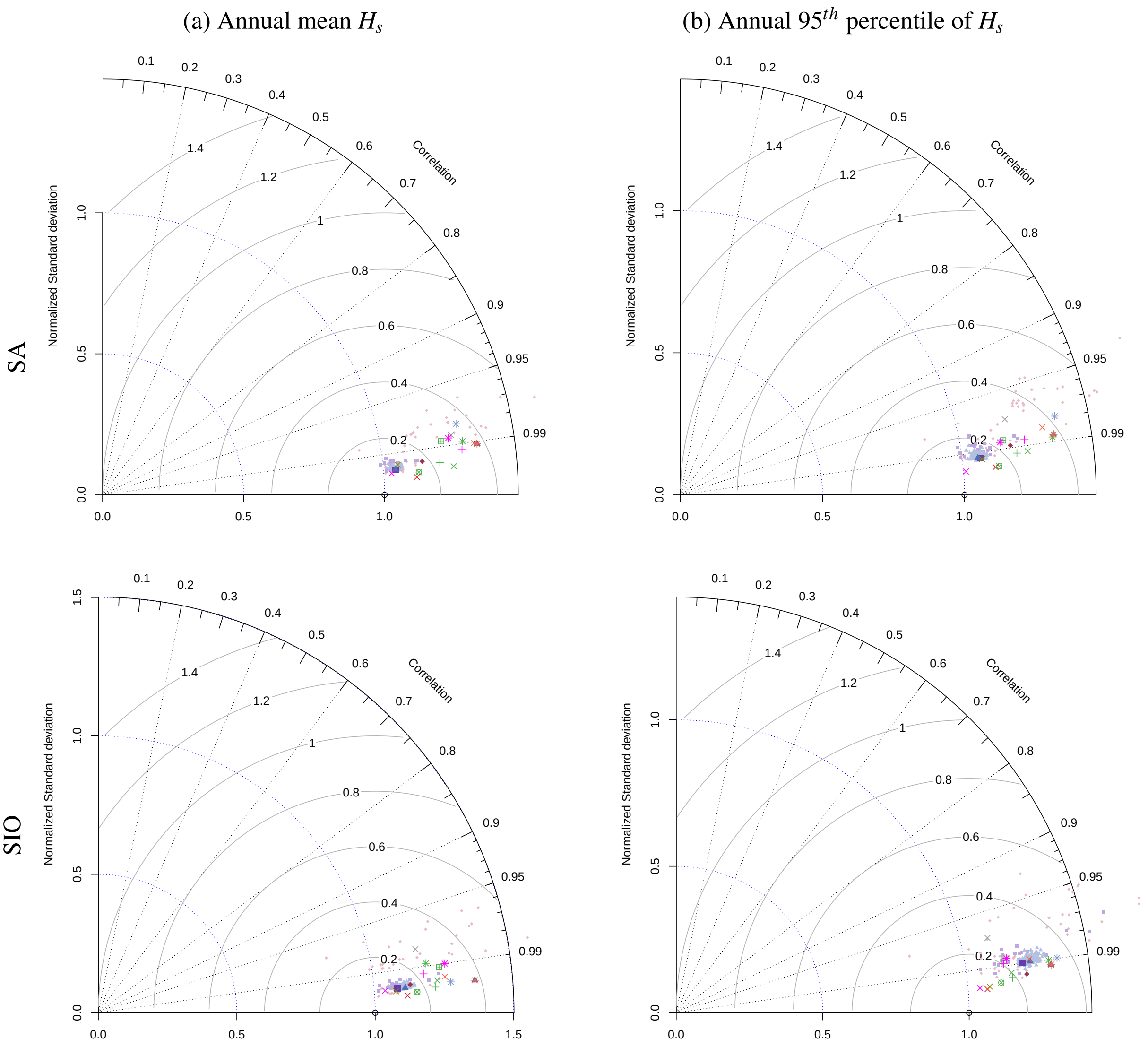

Figure S7. Normalized Taylor diagram for the climatological mean of the annual mean (a) and the annual 95th percentile (b) of H_s for the indicated regions (see Figure S1). ERA5 is used as reference. Legend shown in Figure S2.

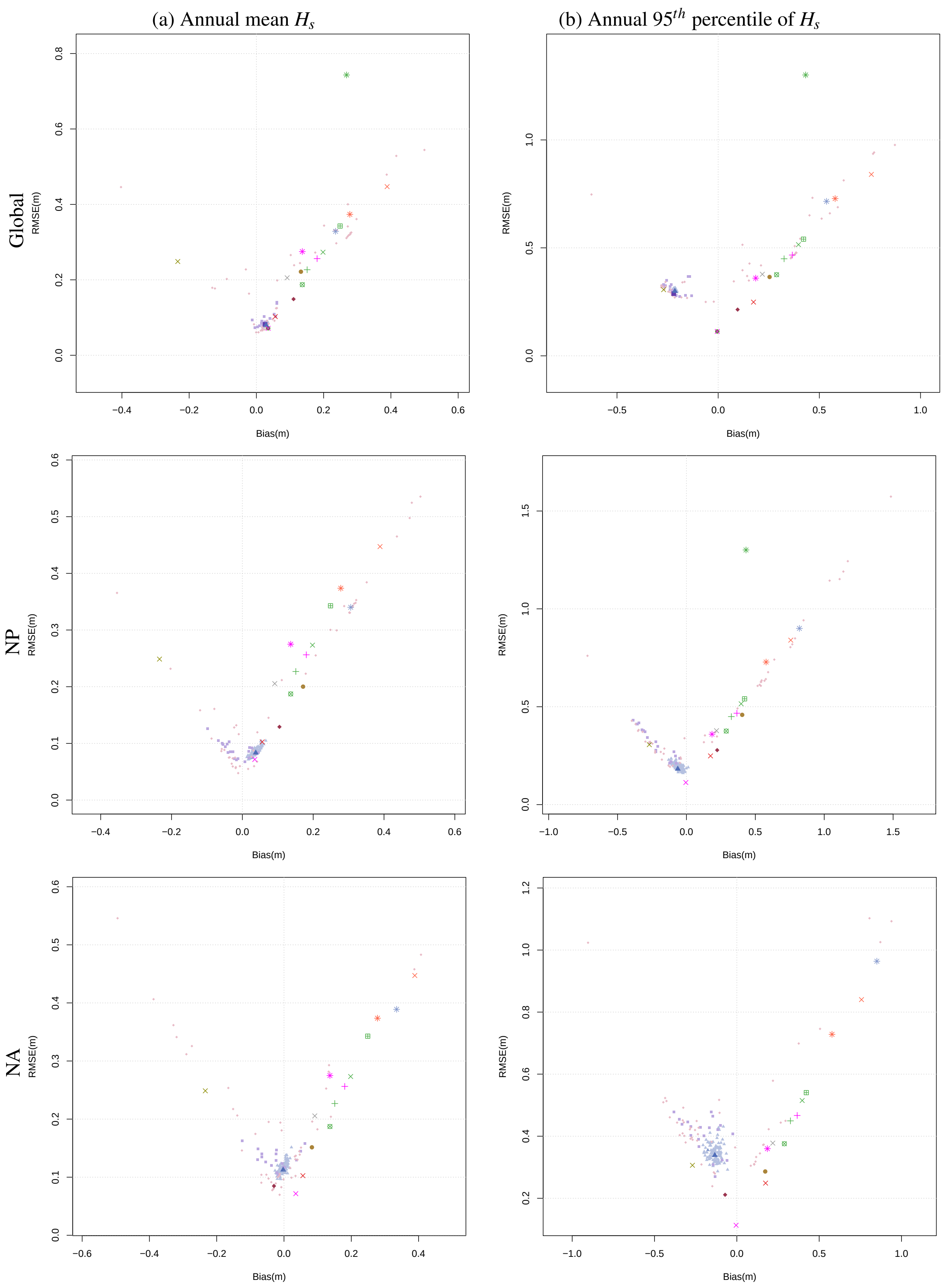

Figure S8. RMSE vs. Bias (in m) for the climatological mean of the annual mean (a) and annual 95th percentile (b) of H_s for the indicated regions (see Figure S1). ERA5 is used as reference. Legend shown in Figure S2.

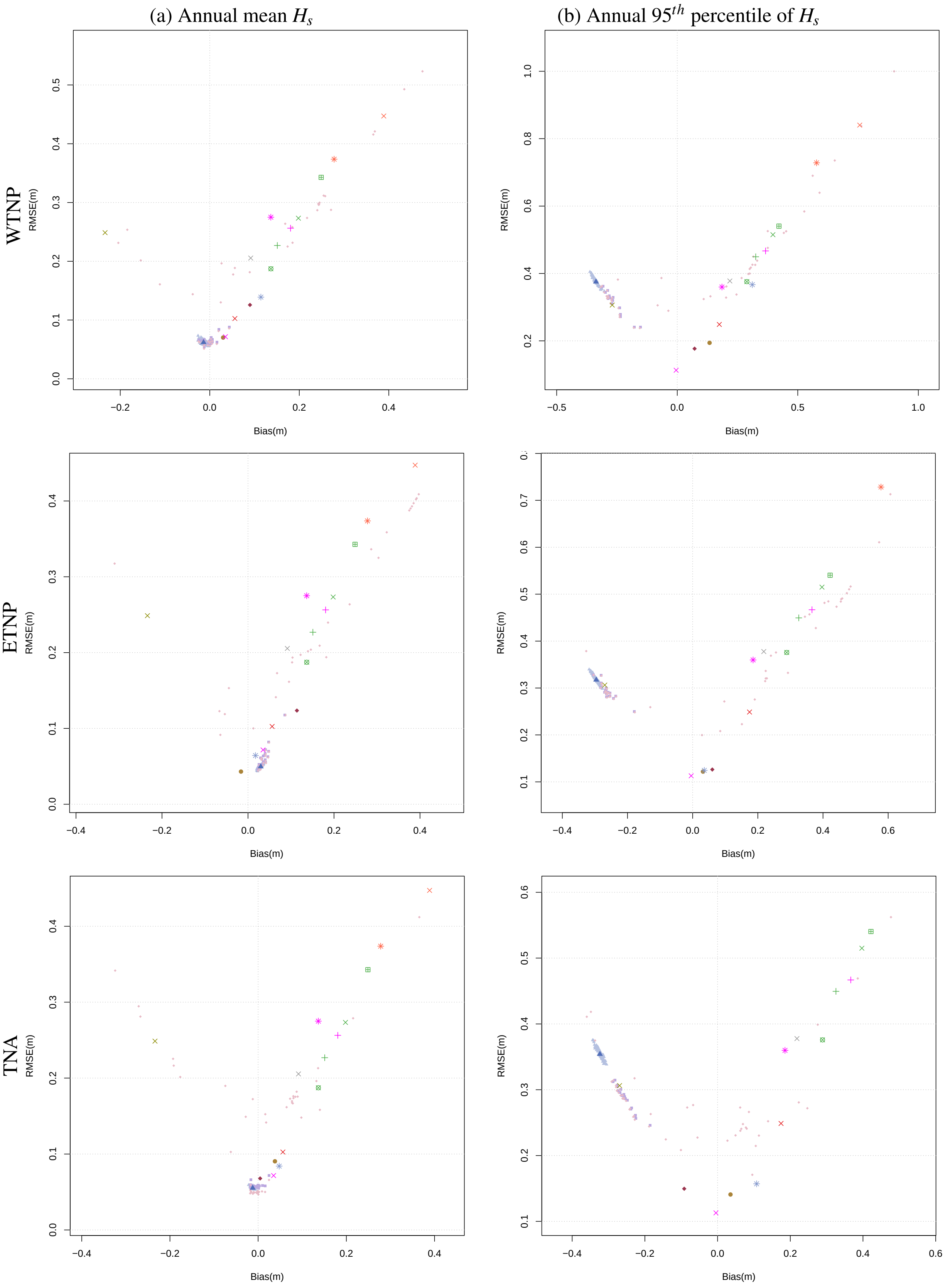

Figure S9. RMSE vs. Bias (in m) for the climatological mean of the annual mean (a) and annual 95th percentile (b) of H_s for the indicated regions (see Figure S1). ERA5 is used as reference. Legend shown in Figure S2.

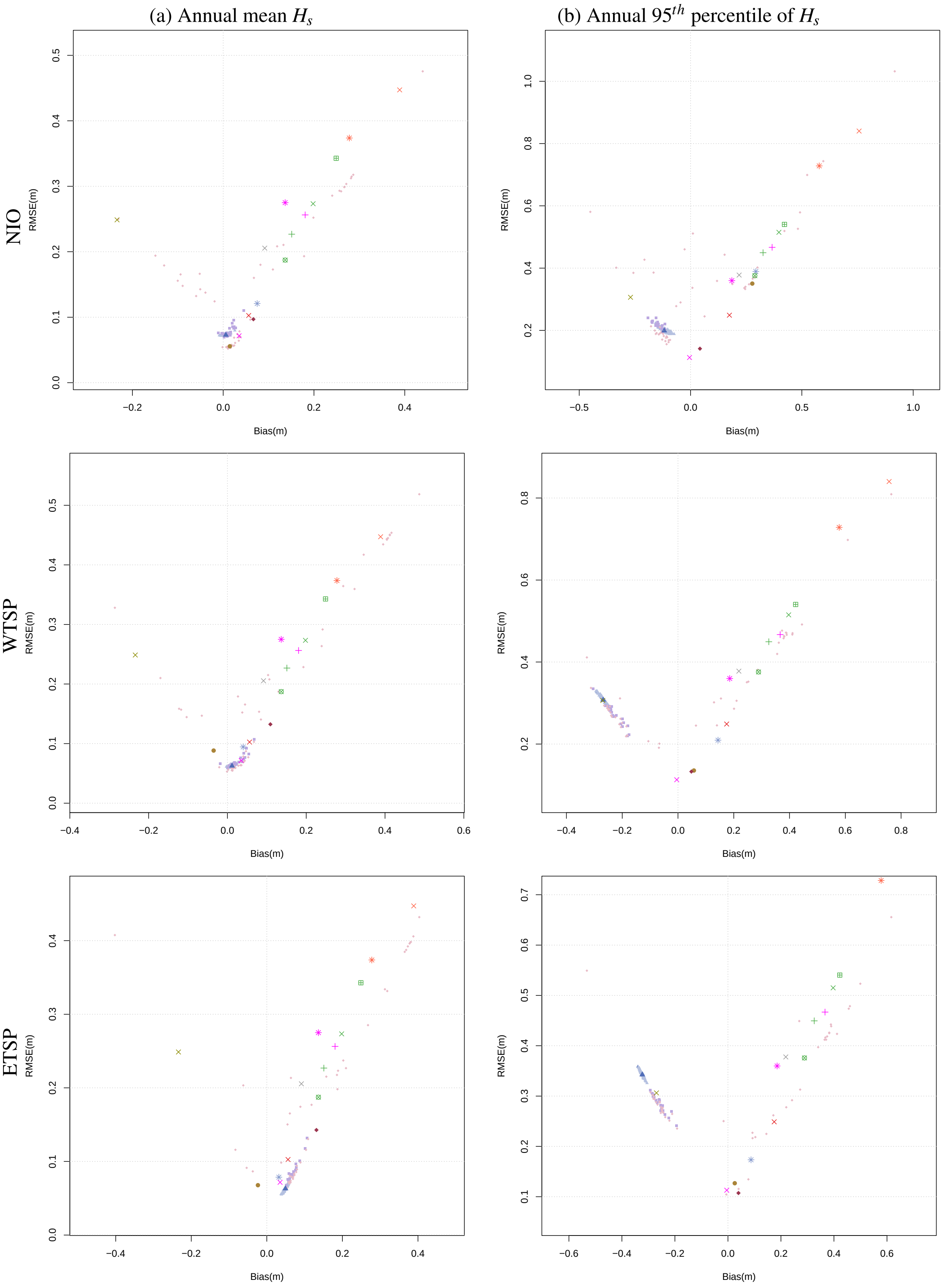

Figure S10. RMSE vs. Bias (in m) for the climatological mean of the annual mean (a) and annual 95th percentile (b) of H_s for the indicated regions (see Figure S1). ERA5 is used as reference. Legend shown in Figure S2.

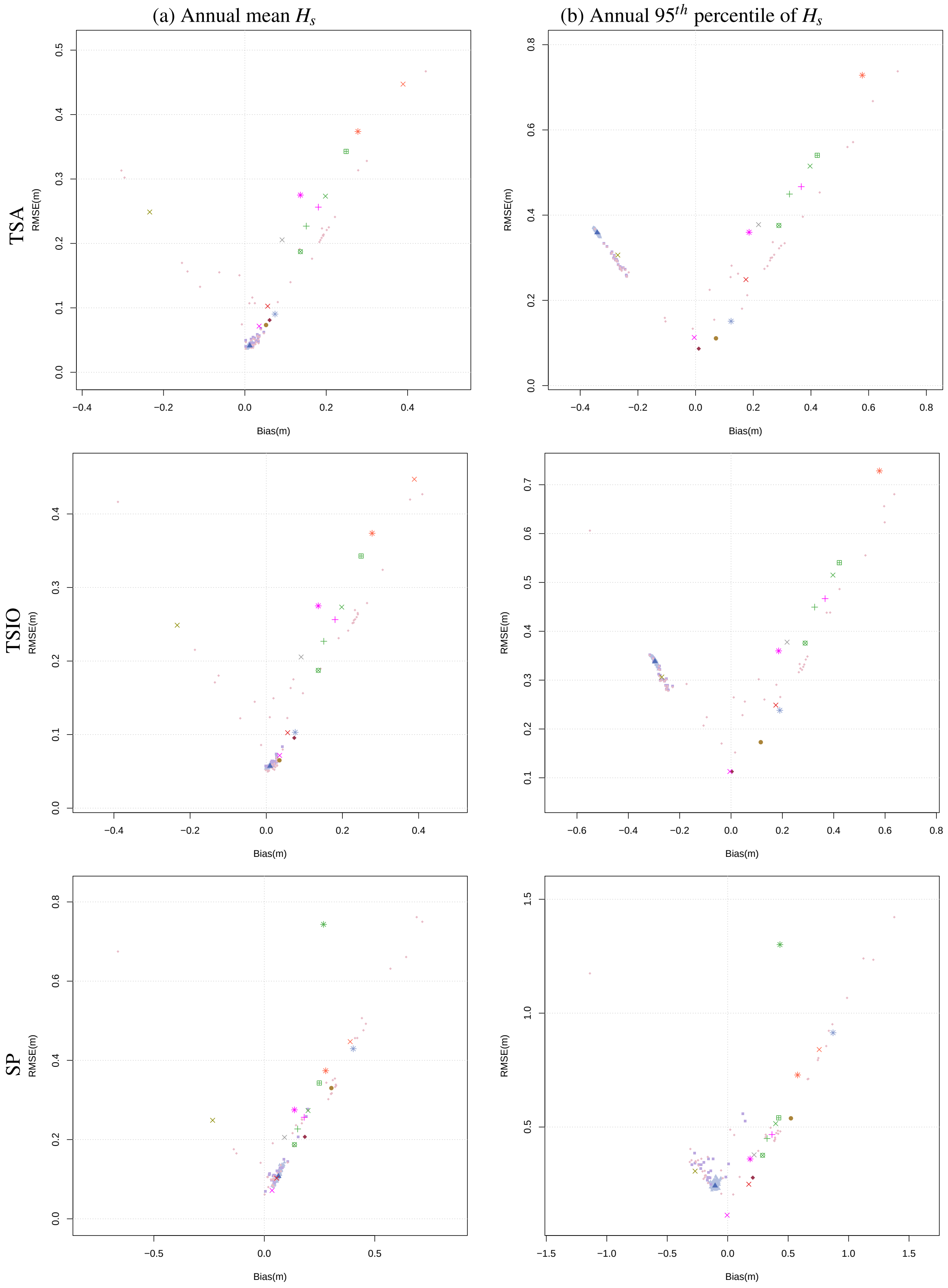

Figure S11. RMSE vs. Bias (in m) for the climatological mean of the annual mean (a) and annual 95th percentile (b) of H_s for the indicated regions (see Figure S1). ERA5 is used as reference. Legend shown in Figure S2.

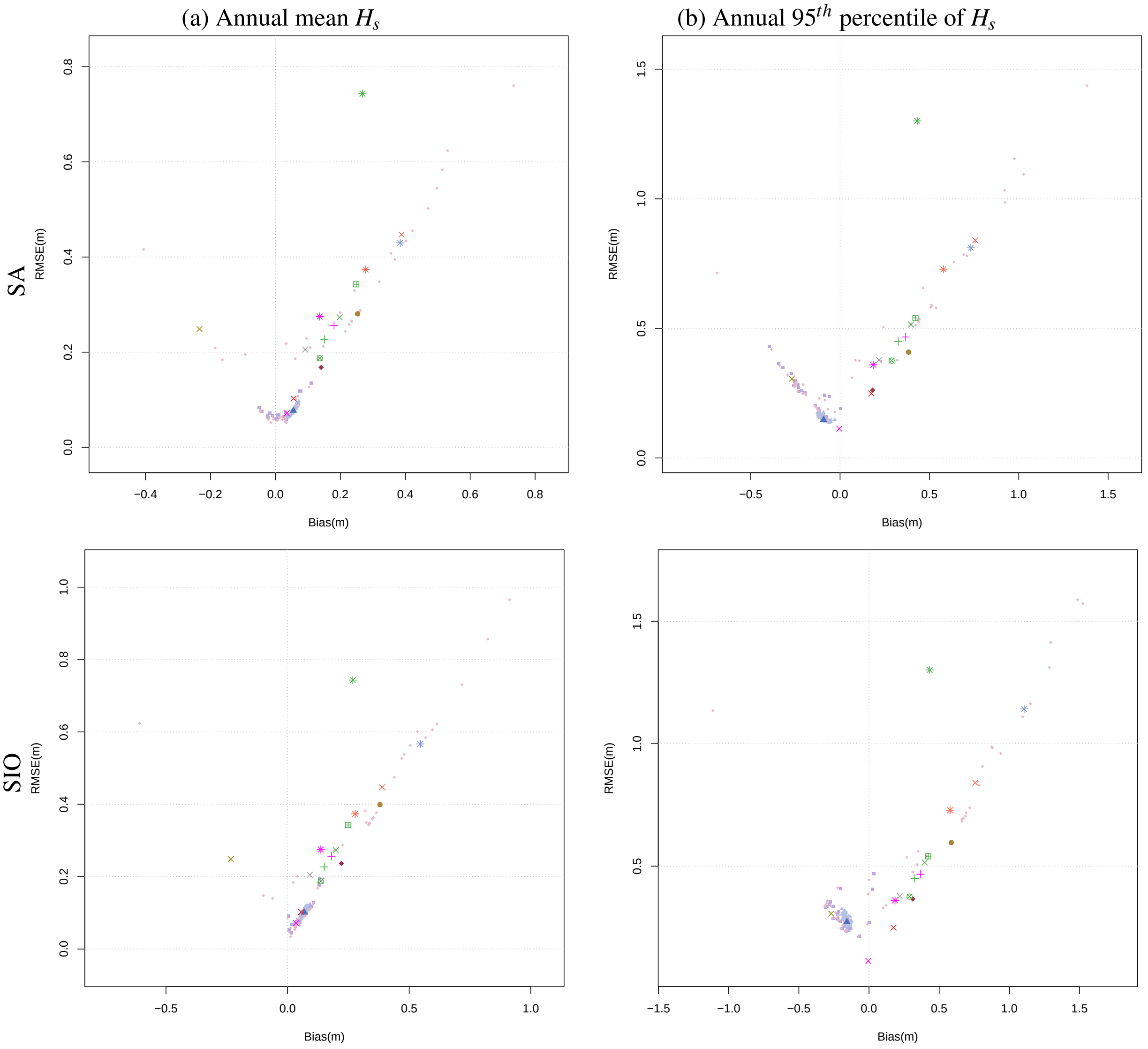

Figure S12. RMSE vs. Bias (in m) for the climatological mean of the annual mean (a) and annual 95th percentile (b) of H_s for the indicated regions (see Figure S1). ERA5 is used as reference. Legend shown in Figure S2.

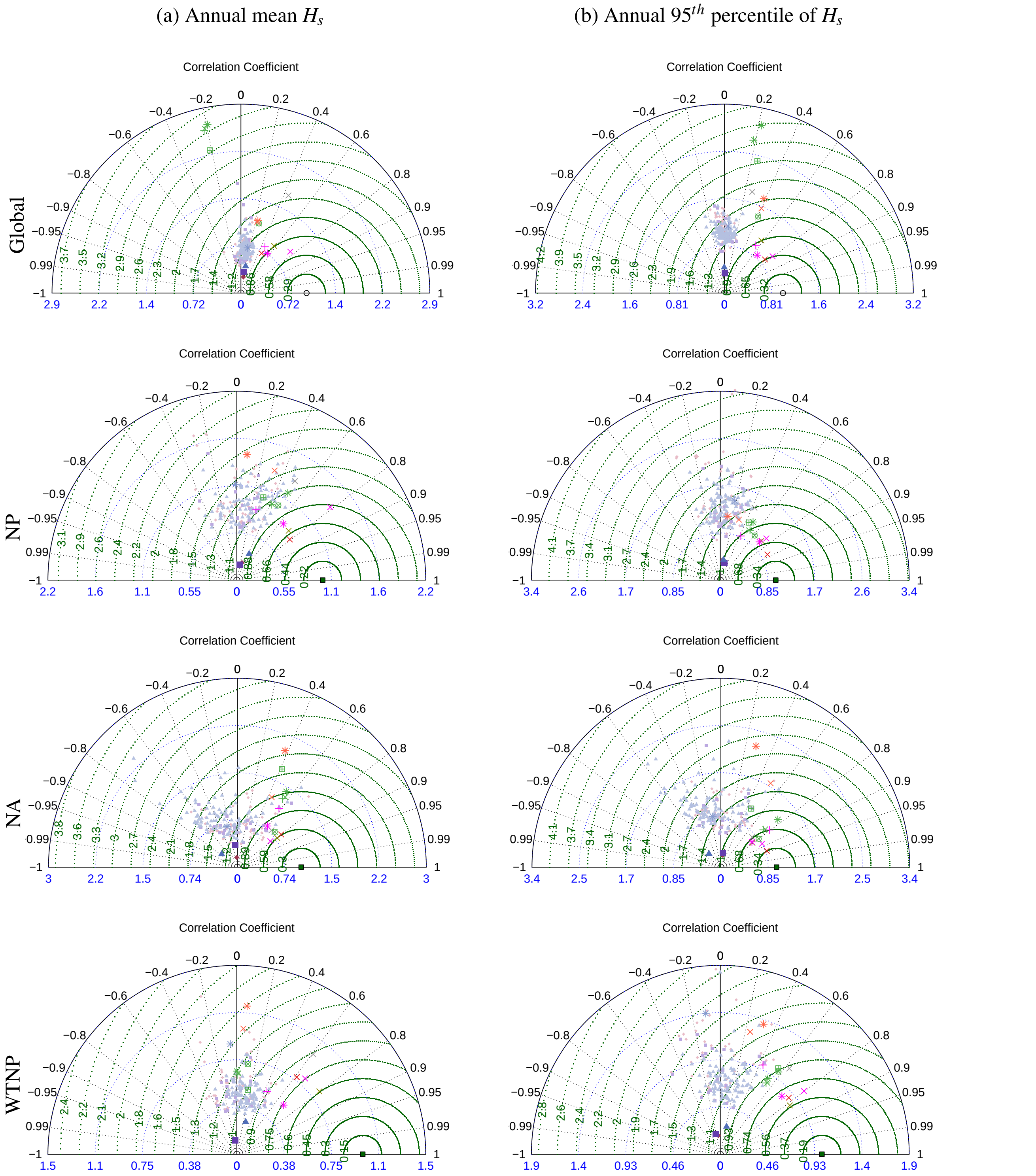

Figure S13. Normalized Taylor diagram for the annual mean (a) and annual 95th percentile (b) of H_s trends for the indicated regions (see Figure S1). ERA5 is used as reference. Legend shown in Figure S2.

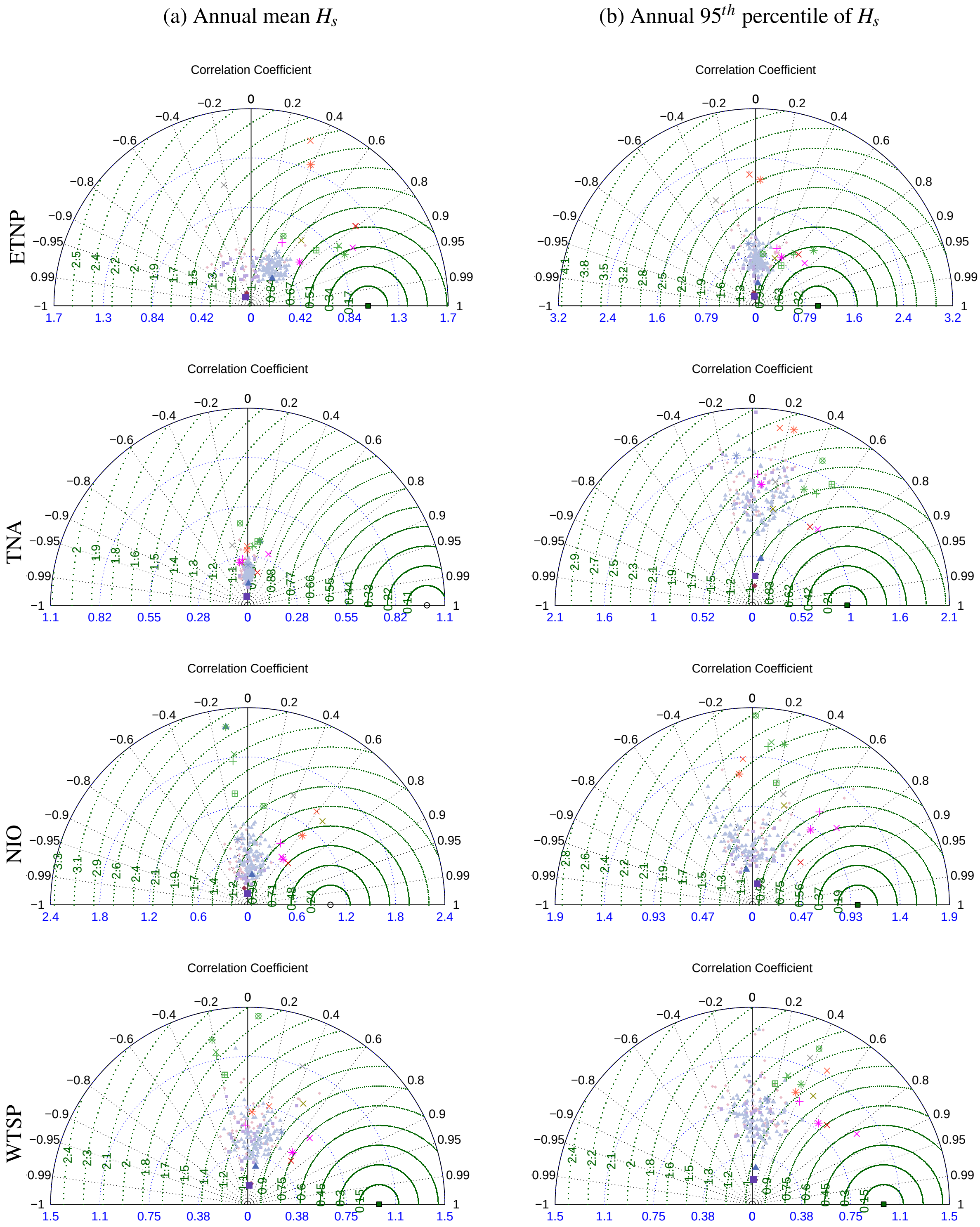

Figure S14. Normalized Taylor diagram for the annual mean (a) and annual 95th percentile (b) of H_s trends for the indicated regions (see Figure S1). ERA5 is used as reference. Legend shown in Figure S2.

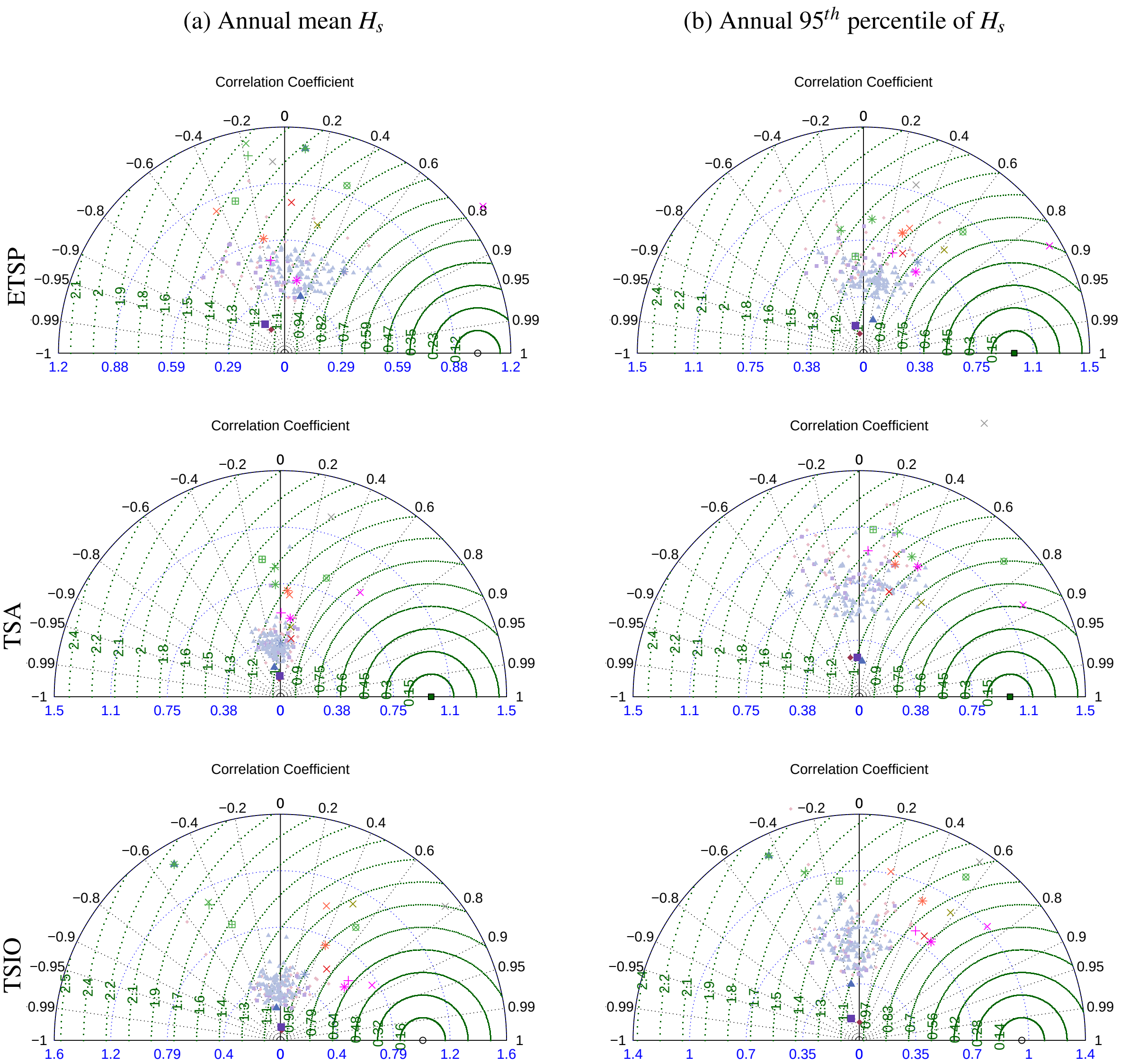

Figure S15. Normalized Taylor diagram for the annual mean (a) and annual 95th percentile (b) of H_s trends for the indicated regions (see Figure S1). ERA5 is used as reference. Legend shown in Figure S2.

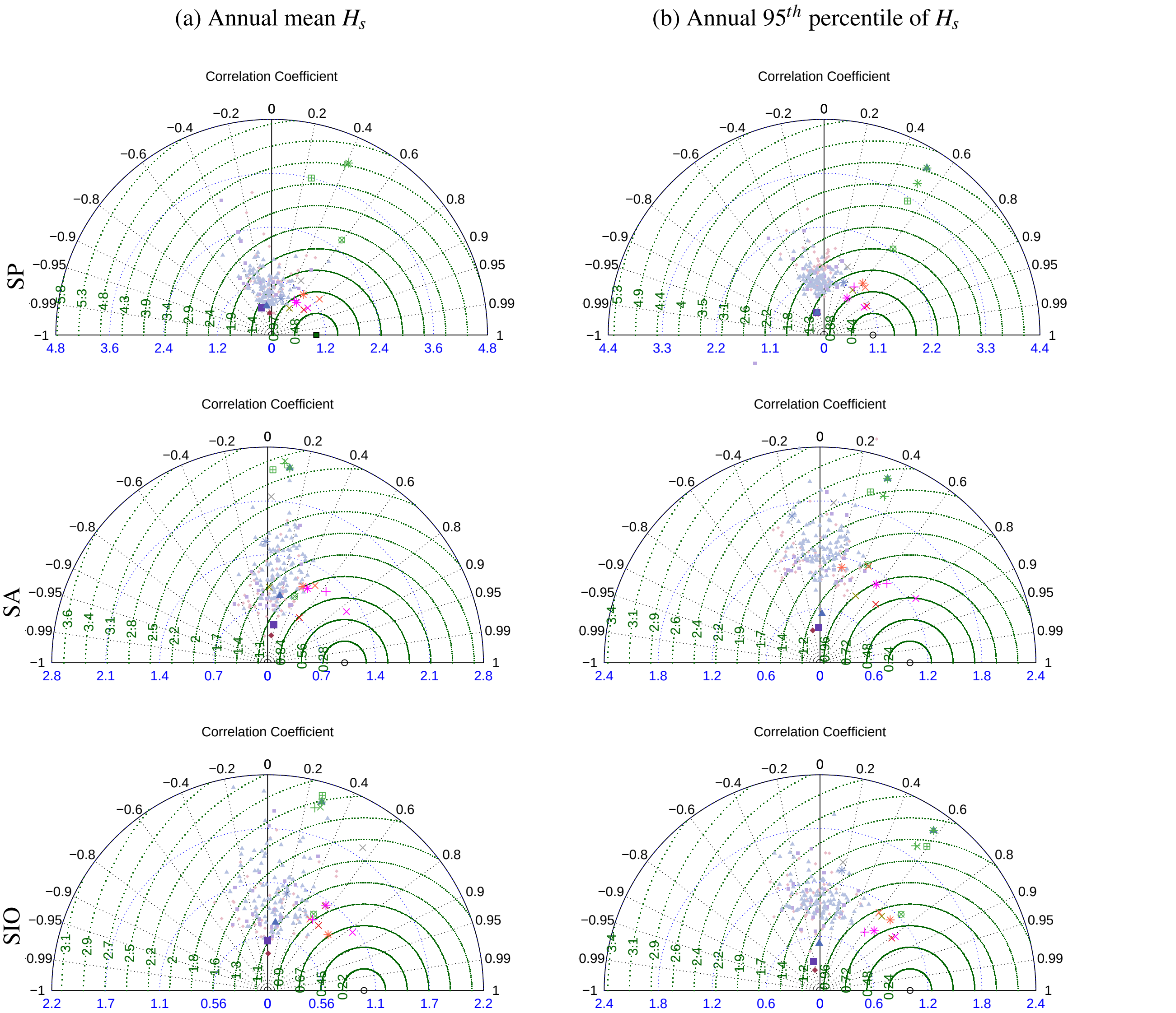

Figure S16. Normalized Taylor diagram for the annual mean (a) and annual 95th percentile (b) of H_s trends for the indicated regions (see Figure S1). ERA5 is used as reference. Legend shown in Figure S2.

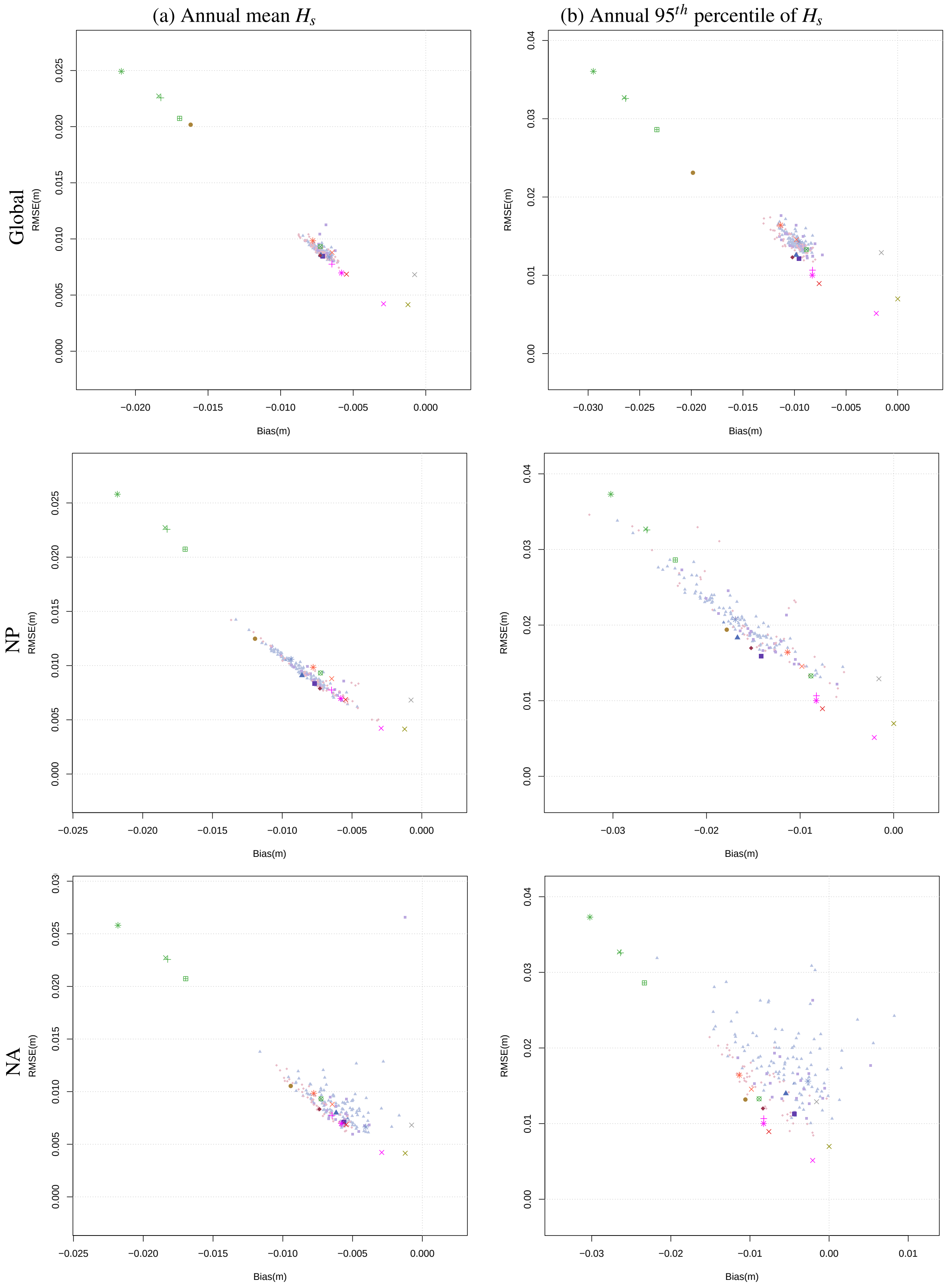

Figure S17. RMSE vs. Bias (in m) for the trend of the annual mean (a) and annual 95th percentile (b) of H_s for the indicated regions (see Figure S1). ERA5 is used as reference. Legend shown in Figure S2.

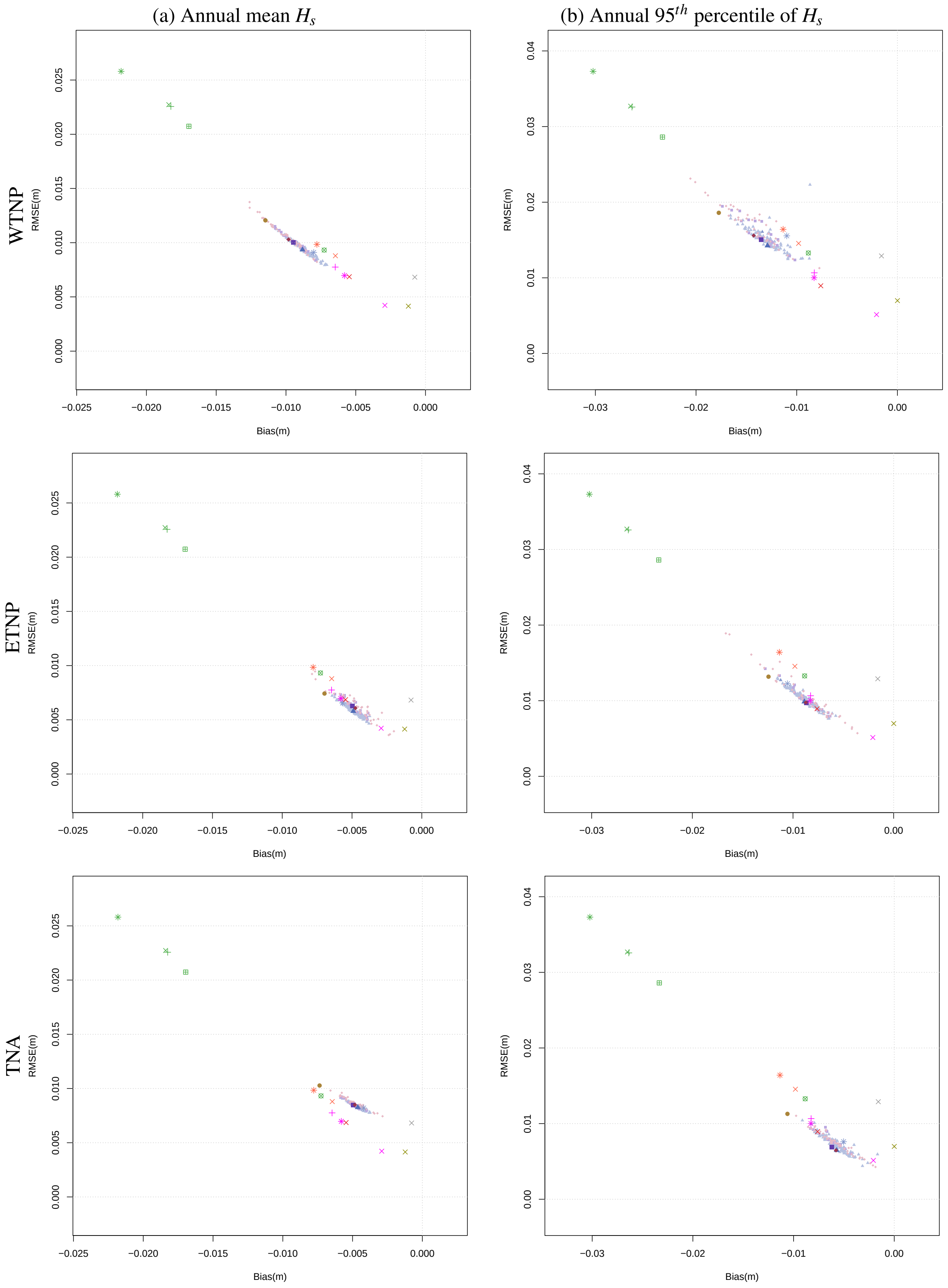

Figure S18. RMSE vs. Bias (in m) for the trend of the annual mean (a) and annual 95th percentile (b) of H_s for the indicated regions (see Figure S1). ERA5 is used as reference. Legend shown in Figure S2.

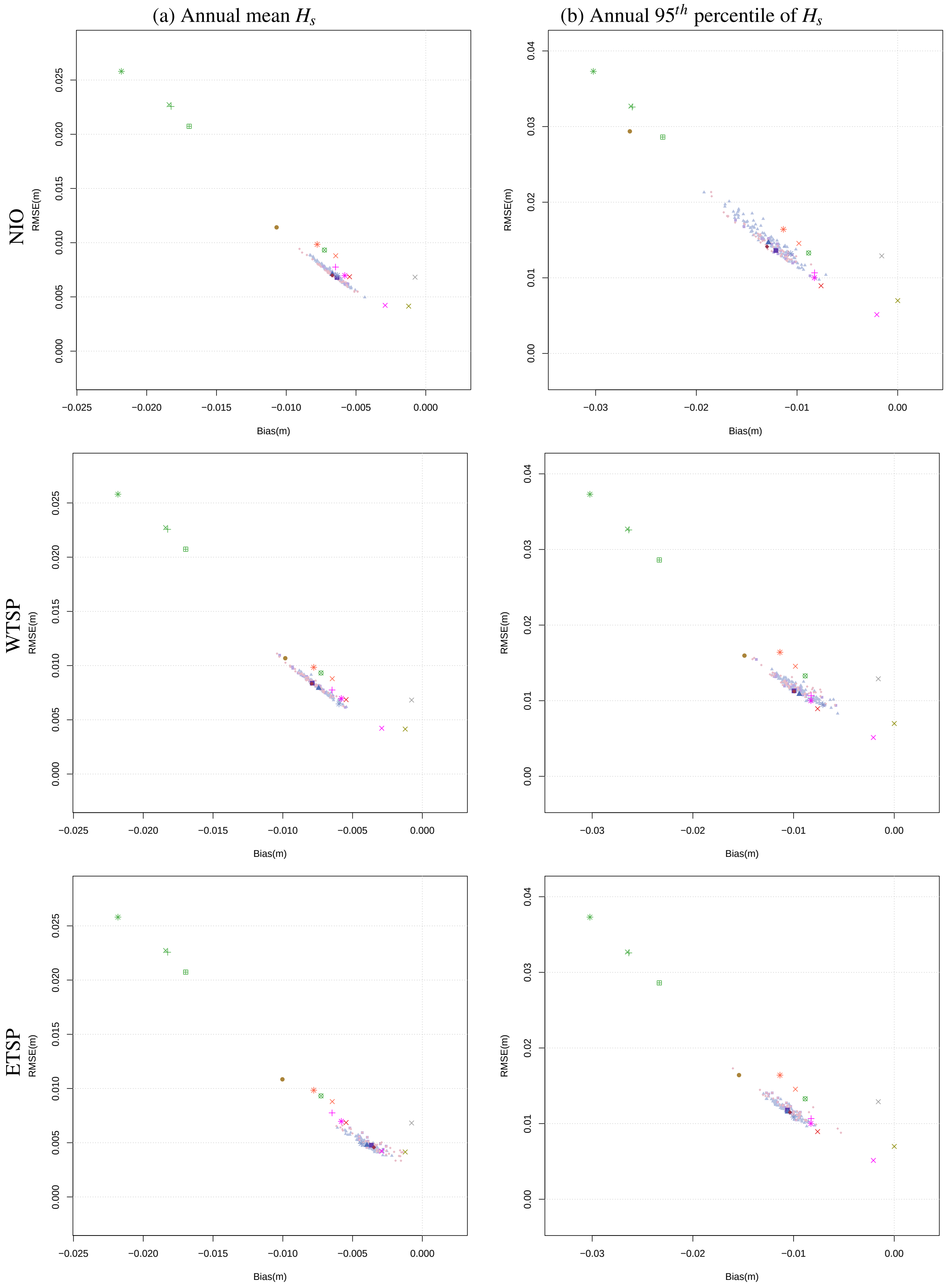

Figure S19. RMSE vs. Bias (in m) for the trend of the annual mean (a) and annual 95th percentile (b) of H_s for the indicated regions (see Figure S1). ERA5 is used as reference. Legend shown in Figure S2.

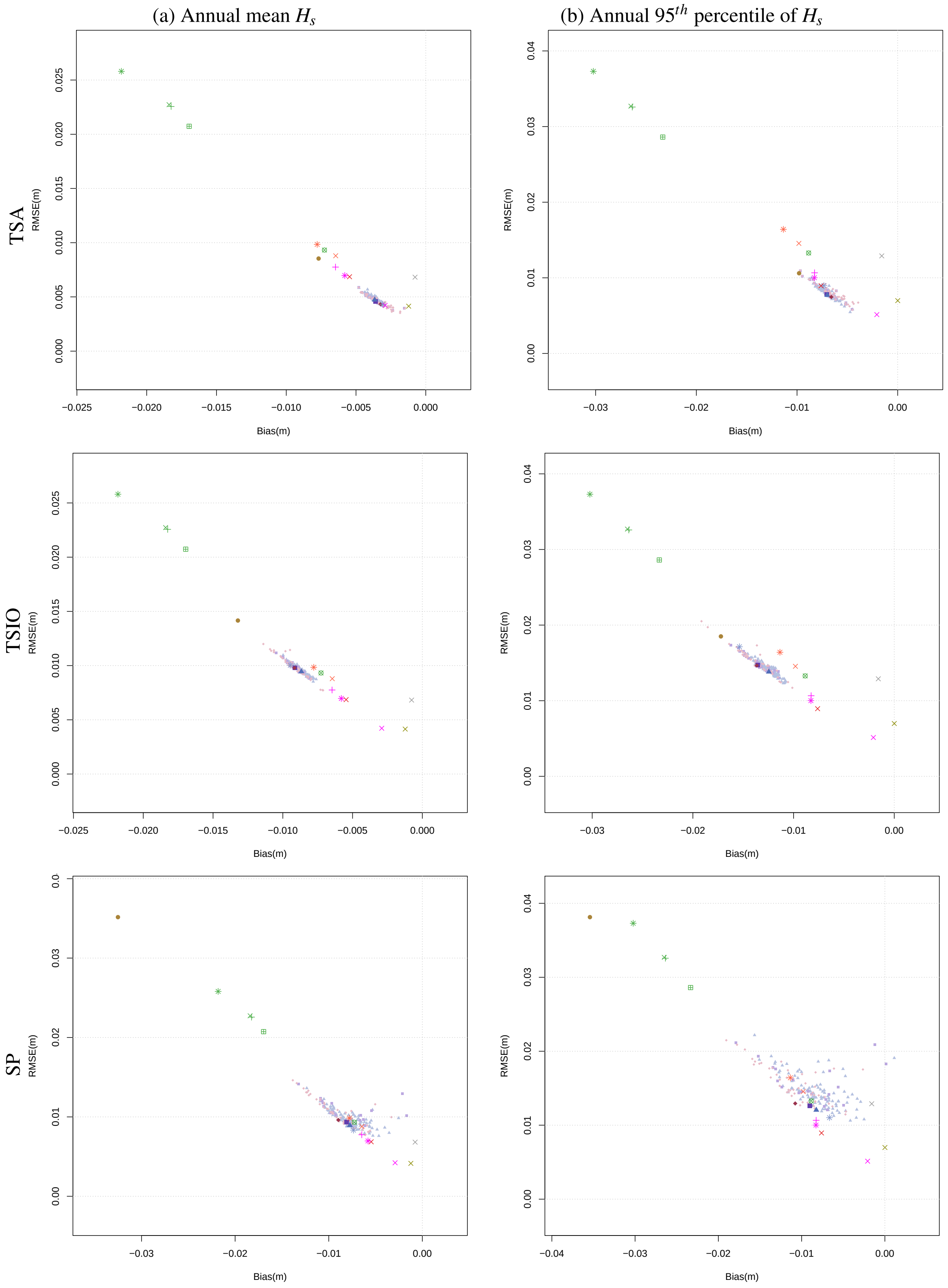

Figure S20. RMSE vs. Bias (in m) for the trend of the annual mean (a) and annual 95th percentile (b) of H_s for the indicated regions (see Figure S1). ERA5 is used as reference. Legend shown in Figure S2.

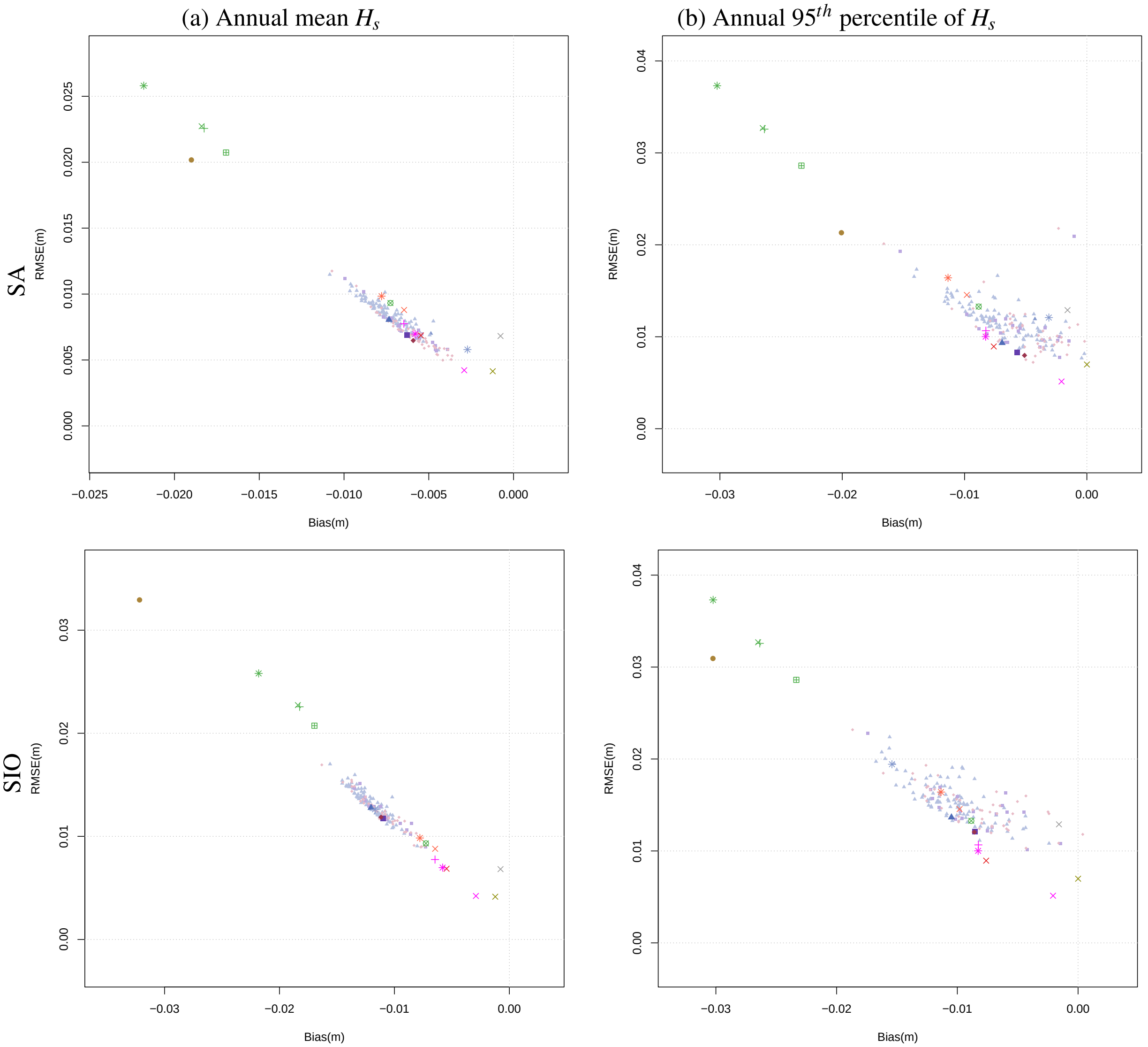

Figure S21. RMSE vs. Bias (in m) for the trend of the annual mean (a) and annual 95th percentile (b) of H_s for the indicated regions (see Figure S1). ERA5 is used as reference. Legend shown in Figure S2.
